# Effects of ambient temperature on growth performance, slaughter traits, meat quality and serum antioxidant function in Pekin duck

**DOI:** 10.3389/fvets.2024.1363355

**Published:** 2024-03-06

**Authors:** Congcong Xu, Dongyue Sun, Yi Liu, Ziyi Pan, Zichun Dai, Fang Chen, Rihong Guo, Rong Chen, Zhendan Shi, Shijia Ying

**Affiliations:** ^1^Institute of Animal Science, Jiangsu Academy of Agricultural Sciences, Nanjing, China; ^2^College of Animal Science and Technology, Beijing University of Agricultural, Beijing, China; ^3^College of Animal Science and Technology, Nanjing Agricultural University, Nanjing, China; ^4^College of Life Sciences, Jiangsu University, Zhenjiang, China; ^5^Key Laboratory of Crop and Animal Integrated Farming, Ministry of Agriculture, Nanjing, China

**Keywords:** Pekin duck, temperature, growth performance, slaughtering traits, meat quality, antioxidant function

## Abstract

The present study investigated the effects of temperature on growth performance, slaughtering traits, meat quality and antioxidant function of Pekin ducks from 21–42 d of age. Single factor analysis of variance was used in this experiment, 144 21 d-old Pekin ducks were randomly allotted to 4 environmentally controlled chambers: T20 (20°C), T23 (23°C), T26 (26°C) and T29 (29°C), with 3 replicates in each group (12 ducks in each replicate), the relative humidity of all groups is 74%. During the 21-day trial period, feed and water were freely available. At 42 d, the BW (body weight) and ADG (average daily gain) of T26 were significantly lower than T20 (*p* < 0.05), and the T29 was significantly lower than T20 and T23 (*p* < 0.05). The ADFI (average daily feed intake) of T26 and T29 were significantly lower than T20 and T23 (*p* < 0.05). Compared to the T29, the T20 showed a significant increase oblique body length and chest width, and both the keel length and thigh muscle weight significantly increased in both the T20 and T23, while the pectoral muscle weight increased significantly in other groups (*p* < 0.05). The cooking loss of the T29 was the lowest (*p* < 0.05). The T-AOC (total antioxidant capacity) of T29 was significantly higher than the other groups (*p* < 0.05), the SOD (superoxide dismutase) in the T29 was significantly higher than the T23 and T26 (*p* < 0.05). In conditions of 74% relative humidity, the BW and ADFI of Pekin ducks significantly decrease when the environmental temperature exceeds 26°C, and the development of body size and muscle weight follows this pattern. The growth development and serum redox state of Pekin ducks are more ideal and stable at temperatures of 20°C and 23°C.

## 1 Introduction

China stands as the world’s foremost market for duck production and consumption, with approximately 5.59 billion ducks produced in the country in 2022. Among these, Pekin ducks constitute 80%, while other breeds make up the remaining 20%. Notably, the predominant strains within the Pekin duck category employed for meat production include “Grassland duck,” “Zhongxin duck,” and “Z-type Pekin duck” (1–4). The methods of waterfowl farming are continually evolving, and indoor dryland farming has been extensively utilized in the duck industry. The change of environmental temperature and humidity, together with a variety of physiological situations including age, fasting and food intake, stress circumstances and inflammation status induce changes in internal temperature which are followed by changes in body surface temperature (5, 6). The pivotal role of ambient temperature in the indoor environment of dryland duck farming has been widely acknowledged (7–10). In addition, as the global trend of climate warming becomes increasingly apparent, research on the impact of temperature on the breeding industry is pressing. The economic efficiency of the poultry industry is closely linked to the growth rate and mortality rate in poultry production systems, and ambient temperature plays a decisive role in the health and growth of animals (11–15).

Poultry are susceptible to temperature due to factors such as rapid metabolic rate, increased basal metabolic heat production, and rapid growth and production. Heat dissipation is an important challenge for poultry because they lack sweat glands. In addition, feathers and fluff have a good insulation effect, which makes the heat dissipation of poultry more difficult. To balance the increased metabolic heat production, the body secretes more leptin to stimulate the hypothalamus to reduce the animal’s appetite, thereby reducing food intake (12, 16, 17). Animals will increase feed intake and accelerate the body’s heat production rate to combat hypothermia in cold environments, prolonged hypothermia affects animal performance and causes disease and thus increased mortality (11, 18, 19). Current research on environmental temperature in meat poultry farming mainly focused on broilers and geese, with fewer reports on ducks. Chronic heat stress induced by elevated environmental temperatures can diminish broiler chickens’ growth performance, pectoral muscle yield and meat quality (20–23), as well as lead to physiological changes in intestinal morphology and oxidative damage (24, 25). When the temperature exceeds 25.19°C, the growth performance and fat deposition of geese are inhibited, and the morphology of the duodenum is more susceptible to damage (26). Sun et al. estimated the upper critical temperature for the optimal growth of Pekin ducks through a model. The BW, pectoral muscle weight, thigh muscle weight, pectoral muscle ratio, abdominal fat and abdominal fat ratio of Pekin ducks all show a linear or quadratic decrease with increasing temperature (27). High ambient temperatures also increase serum protein cortisol levels in breeding ducks and reduce growth and reproductive performance (14).

In our previous research, we found that the environment with 74% relative humidity is more favorable for Pekin ducks (3). However, most of the current research on the impact of temperature on Pekin ducks is based on a 60% humidity environment (28). Moreover, many reports indicate that temperatures above 30°C have significant adverse effects on poultry (29). Therefore, this study will investigate the effects of 20–29°C on Pekin ducks on the basis of 74% humidity. We will further explore the optimal environmental temperature range for the growth of Pekin ducks, providing empirical support and theoretical basis for the environmental parameter regulation in the waterfowl dry farming model.

## 2 Materials and methods

### 2.1 Animals, ethics and experimental design

The experimental procedures were approved by the Research Committee of Jiangsu Academy of Agricultural Sciences and conducted with adherence to the Regulations for the Administration of Affairs Concerning Experimental Animals (Decree No. 63 of the Jiangsu Academy of Agricultural Science on July 8, 2014). All Pekin ducks were purchased from China Danyang Yantian Poultry Breeding Cooperative Society (Danyang, China). 144 21 d-old ducks were in good health and randomly divided into 4 environmentally controlled chambers with ambient temperatures set at 20°C, 23°C, 26°C and 29°C. In each chamber, 36 ducks were divided randomly into 3 raised wire-floor pens of 12 ducks each. All ducks had similar initial body weights at the start of the experiment. All environmentally controlled chambers have a relative humidity of 74%, with food and drinking water available *ad libitum*, and all ducks were kept in environmentally controlled chamber for 21 d.

Beijing Kulan Technology Co. constructed the environmentally controlled chambers. The temperature and humidity were automatically adjusted by the control room according to set parameters (temperature accuracy ±1°C, humidity accuracy ±5%), with 20 h of light and 4 h of dark. The LED-tubes with a color temperature of 6,000 K and a light intensity of 15 lux was chosen to be light source (30). All experimental ducks were reared in 4 identical enclosed chambers with dimensions of 4 m × 2.5 m × 2.6 m. The walls and floors of the chambers were constructed using insulation materials. All ducks were raised on polyethylene prefabricated leakage dung plates. The leakage dung plates were 60 cm above the ground and fixed with galvanized steel frames. Dry rice husks, 20 cm thick, were spread on the ground. Temperature and humidity sensors were set 60 cm above the manure leakage board to monitor room temperature and humidity in real time.

The ducks were allowed to feed and drink freely during this trial. The water was provided through nipple drinkers, and the height was adjusted periodically according to the ducks’ growth. Feeding feed complies with feed hygiene standard GB/13078–2017 (31), and the nutritional level complies with meat duck feeding standard NY/T2122-2012 (32). The composition of the feed is detailed in Table 1.

**Table 1:** Composition and nutrient levels of basal diet (%, air-dry basis) in 1–14 days and 15–42 days.

Ingredient	Dietary energy (0–14 days)	Dietary energy (15–42 days)	Calculated nutrition levels	Dietary energy (1–14 days)	Dietary energy (15–42 days)
Corn	62.10	68.5	ME (Kcal/kg)	2,900	2,950
Wheat	5.22	3.02	Crude protein (%)	20.01	17.52
Soybean meal	28.60	24.30	Ca (%)	0.90	0.85
Rapeseed meal	0.00	0.60	Available P (%)	0.42	0.40
Limestone	0.93	0.90	Digestible lysine (%)	0.98	0.82
CaHPO4 (2H2O)	1.85	1.72	Digestible methionine (%)	0.47	0.37
NaCl	0.34	0.33	Digestible Met + Cys (%)	0.75	0.64
Choline chloride	0.15	0.15	Digestible threonine (%)	0.65	0.56
Premix*	0.23	0.23	Digestible tryptophan (%)	0.28	0.19
DL-methionine	0.24	0.12			
L-lysine·HCl	0.20	0.10			
L-tryptophan	0.08	0.02			
L-threonine	0.06	0.01			
Total	100	100			

*Supplied per kilogram diet: vitamin A, 9,000 IU; vitamin D3, 2,000 IU; vitamin E, 10 IU; vitamin B1, 2 mg; vitamin B2, 4.8 mg; pantothenic acid, 20 mg; vitamin B12, 0.02 mg; folic acid, 1 mg; niacin 50 mg; Cu (CuSO4·5H2O), 8 mg; Fe (FeSO4·7H2O), 60 mg; Zn (ZnSO4·7H2O), 60 mg; Se (NaSeO3), 0.3 mg; I (KI), 0.4 mg.

Daily feed intake was recorded throughout the experiment, and the weight of the test ducks and remaining feed intake were recorded every 3 days. The ADG, ADFI and F/G (feed-to-weight) were calculated according to the following equations:

$$\begin{array}{l} ADG\;\left(g/d\right)=\mathrm{Total}\;\mathrm{stage}\;\mathrm{Weight}\;\mathrm{gain}\;\left(g\right)/\\ \;\mathrm{Number}\;\mathrm{of}\;\mathrm{feeding}\;\mathrm{days}\;\left(d\right)\text{.} \end{array}$$

$$\begin{array}{l} \mathrm{ADFI}\;\left(g/d\right)=\mathrm{Total}\;\mathrm{stage}\;\mathrm{feed}\;\mathrm{intake}\;\left(g\right)/\\ \;\mathrm{Number}\;\mathrm{of}\;\mathrm{feeding}\;\mathrm{days}\;\left(d\right)\text{.} \end{array}$$

$$\begin{array}{l} F/G\;\left(g/g\right)=\mathrm{Average}\;\mathrm{daily}\;\mathrm{feed}\;\mathrm{intake}\;\left(g\right)/\\ \;\mathrm{Average}\;\mathrm{daily}\;\mathrm{weight}\;\mathrm{gain}\;\left(g\right)\text{.} \end{array}$$

### 2.2 Measurement of body size traits and slaughtering traits

At 42 days of age, 3 ducks were randomly selected from each replicate for slaughter in each treatment group. Determine body size and slaughter performance indicators in accordance with NY/T 823–2020 Poultry Production Performance Terminology and Measurement Methods (33).

Live fasting weights of ducks weighed using electronic scales. Live duck immobilized by unarmed head grasp and subjected to body size measurements. Oblique body length, Keel length of live ducks were measured using a tape measure, chest depth, chest width, and dorsal breadth of live ducks were measured using vernier calipers. After slaughtering, a precision electronic scale was used to measure the weights of liver, spleen, pectoral muscle, thigh muscle and abdominal fat of the test ducks. Tissue samples of liver, spleen, abdominal fat, pectoral muscle and thigh muscle were immediately collected in freezing tubes, placed in a liquid nitrogen tank for temporary storage then stored at −80°C. Liver index, spleen index, pectoral muscle rate, thigh muscle rate and abdominal fat rate were calculated according to the following equations:

$$\mathrm{Liver}\;\mathrm{index}\;\left(\%\right)=\mathrm{Liver}\;\mathrm{weight}\;\left(g\right)/\mathrm{Live}\;\mathrm{weight}\;\left(g\right)\times 100\%\text{.}$$

$$\mathrm{Spleen}\;\mathrm{index}\;\left(\%\right)=\mathrm{Spleen}\;\mathrm{weight}\;\left(g\right)/\mathrm{Live}\;\mathrm{weight}\;\left(g\right)\times 100\%\text{.}$$

$$\begin{array}{l} \mathrm{Abdominal}\;\mathrm{fat}\;\mathrm{ratio}\;\left(\%\right)=\mathrm{Abdominal}\;\mathrm{fat}\;\mathrm{weight}\;\left(g\right)/\\ \;\mathrm{Live}\;\mathrm{weight}\;\left(g\right)\times 100\%\text{.} \end{array}$$

$$\begin{array}{l} \mathrm{Pectoral}\;\mathrm{muscle}\;\mathrm{ratio}\;\left(\%\right)=\mathrm{Pectoral}\;\mathrm{muscle}\;\mathrm{weight}\;\left(g\right)/\\ \;\mathrm{Live}\;\mathrm{weight}\;\left(g\right)\times 100\%\text{.} \end{array}$$

$$\begin{array}{l} \mathrm{Thigh}\;\mathrm{muscle}\;\mathrm{ratio}\;\left(\%\right)=\mathrm{Thigh}\;\mathrm{muscle}\;\mathrm{weight}\;\left(g\right)/\\ \;\mathrm{Live}\;\mathrm{weight}\;\left(g\right)\times 100\%\text{.} \end{array}$$

### 2.3 Meat quality evaluation

At 42 days of age, 3 ducks were randomly selected from each replicate in each treatment group, and the unilateral pectoral muscle was stripped after slaughter, and the meat quality indexes were measured.

#### 2.3.1 Measurement of pH value

The breast muscle obtained from the slaughter was used as the material for measuring the pH value. Three fixed positions were selected on the breast muscle of the experimental ducks. The pH value of each position was determined using the portable meat acidity meter (Testo 206-pH3, Testo SE & Co. KGaA, Lenzkirch, Germany), and the average value was used to represent the pH measurement of the breast muscle.

#### 2.3.2 Meat color measurement

The unilateral pectoral muscle of the test ducks was stripped, and three fixed positions were selected for each pectoral muscle to determine flesh color Luminance L*, redness a*, and yellowness b*, and each index was repeated three times. Each of these measurements was repeated three times using a colorimeter (CR-400, Konica Minolta Sensing, lnc., Tokyo, Japan).

#### 2.3.3 Shear force measurement

A texture analyzer (C-LM3B, Northeast Agricultural University, Haerbin, China) was used to determine the shear force of the cooked breast samples. Three subsamples of 2 cm × 3 cm × 0.5 cm were cut parallel to the muscle fibers, and the average value was taken.

#### 2.3.4 Cooking loss

Pectoral muscles of ducks were cut into pieces of similar shape and size, weighed and sealed. Sealed samples were maintained at a center temperature of 70°C in a Digital display constant temperature water bath (HH-4, Guohua, Changzhou, China) for 15 min and cooled to room temperature. The samples were weighed, and the percentage of weight loss was calculated. Calculation formula: (initial weight -final weight)/initial weight*100%.

#### 2.3.5 Protein content

Protein content in the muscle was determined using an automatic Kjeldahl nitrogen analyzer (VAP500C, Gerhardt, Königswinte, Germany) according to GB 5009.5–2016 (34).

#### 2.3.6 Crude fat

Crude fat was determined using the Soxhlete extraction method ac-cording to GB 5009.6–2016 (35). The instrument used was a crude fat measuring instrument (S2F-06A, Xinjia, Shanghai, China).

#### 2.3.7 Total ash content

The sample was fully charred to a smoke-free state by heating with a low flame, and then placed in a muffle furnace (QDSH-7, YI − HENG, Shanghai, China) and burned at 550 ± 25°C for 4 h. The mixture was cooled to about 200°C, removed and cooled in a desiccator for 30 min. The burn was repeated to a constant weight, and the ash content was calculated, following the GB 5009.4–2010 (36).

### 2.4 Determination of serum antioxidant indices

At 42 days of age, 3 ducks were randomly selected from each replicate in each treatment group. Blood was collected intravenously before slaughter and placed in blood collection vessels without anticoagulant additives. Immediately after blood collection, place it in the refrigerator at 4°C for 1 h. The blood was centrifuged at a low temperature of 3,500 r/min for 15 min, and the serum was separated and stored at −25°C. According to the instructions of the commercial kit, the absorbance value is determined by setting the wavelength of the enzyme marker. MDA (malondialdehyde): wavelength 532 nm; GSH-PX (glutathione peroxidase): wavelength 412 nm; T-AOC (total antioxidant capacity): wavelength 405 nm; SOD (superoxide dismutase): wavelength 450 nm. All kits used were purchased from Nanjing Jiancheng Bioengineering Institute (Nanjing, China).

### 2.5 Statistical analysis

The experimental data were analysed using one-way analysis of variance (ANOVA) of SPSS (version 26.0, SPSS Inc., Chicago, IL, USA), and then the Duncan multiple-comparison test was performed. The data are expressed as “mean ± SEM deviation” and a *p* < 0.05 indicated a significant difference.

## 3 Results

### 3.1 Growth performance

**Figure 1:**
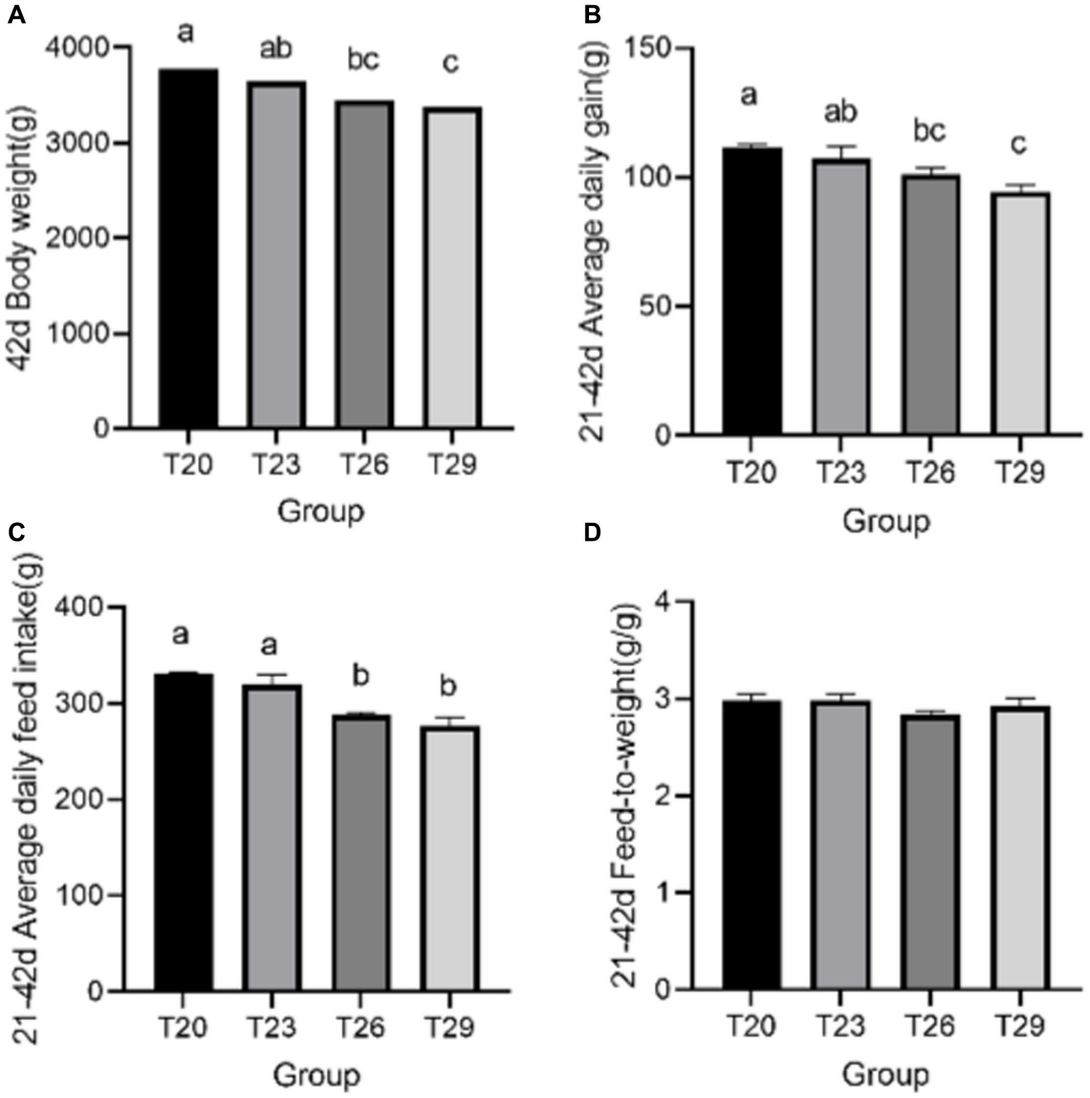
Growth performance. **(A)** 42 day body weight; **(B)** 21–42 day average daily gain; **(C)** 21–42 day average daily feed intake; **(D)** 21–42 day Feed-to-weight. a, b, c Means with different superscripts within the same row differ significantly (*p* < 0.05). No letter indicates that the differences are not significant (*p* > 0.05). Results are means with *n* = 3 per treatment.

At 42 days of age, the BW and ADG of T26 were significantly lower than T20 (*p* < 0.05), and the T29 was significantly lower than T20 and T23 (*p* < 0.05). The ADFI of T26 and T29 were significantly lower than T20 and T23 (*p* < 0.05). And F/G was not significantly different between temperature groups (*p* > 0.05) (Figure 1).

### 3.2 Body size traits and slaughter traits

Compared to the T29, oblique body length of T20 was significantly higher (*p* < 0.05), the keel length and thigh muscle weight of T20 and T23 were significantly higher (*p* < 0.05). The chest width of T20 was significantly higher than T26 and T29 (*p* < 0.05). The live weight, liver weight, spleen weight and pectoral muscle weight were significantly higher in the T20, T23 and T26 (*p* < 0.05). The chest depth, dorsal breadth, abdominal fat weight, liver index, spleen index, abdominal fat ratio, pectoral muscle ratio and thigh muscle ratio were not significantly different between temperature groups (*p* > 0.05) (Table 2).

**Table 2:** Body size and slaughter traits.

Item	Group				SEM	*p*
	T20	T23	T26	T29		
Oblique body length (cm)	32.7^a^	31.9^ab^	31.3^ab^	30.7^b^	0.3	0.032
Keel length (cm)	16.5^a^	16.4^a^	15.6^b^	15.5^b^	0.2	0.015
Chest depth (cm)	62.7	64.6	60.7	61.0	1.8	0.807
Chest width (cm)	63.9^a^	59.2^ab^	54.8^b^	58.0^b^	1.1	0.015
Dorsal breadth (cm)	103.5	103.5	100.9	106.1	2.2	0.877
Pectoral muscle weight (g)	446.5^a^	453.0^a^	464.2^a^	345.3^b^	13.9	<0.01
Pectoral muscle ratio (%)	12.1	12.2	12.7	11.2	0.3	0.175
Thigh muscle weight (g)	335.6^a^	341.8^a^	315.4^ab^	281.7^b^	6.5	<0.01
Thigh muscle ratio (%)	9.06	9.18	8.61	9.14	0.14	0.451
Abdominal fat weight (g)	19.1	21.3	24.4	19.1	1.1	0.281
Abdominal fat ratio (%)	0.52	0.57	0.66	0.62	0.03	0.294
Liver weight (g)	95.3^a^	96.2^a^	87.2^a^	67.0^b^	3.5	<0.01
Liver index	2.57	2.58	2.38	2.14	0.08	0.138
Spleen weight (g)	3.12^a^	2.94^a^	2.73^a^	1.99^b^	0.13	0.01
Spleen index	8.45	7.86	7.49	6.40	0.00	0.143

^a,b,c^Means with different superscripts within the same row differ significantly (*p* < 0.05). No letter indicates that the differences are not significant (*p* > 0.05). Results are means with *n* = 9 per treatment.

### 3.3 Meat quality

The yellowness b* values of the T20 and T26 were significantly lower than the T23 (*p* < 0.05), and the cooking loss of the T29 was significantly lower than the other groups (*p* < 0.05). The measured pH, luminance L*, redness a*, shear force, protein, crude fat and ash did not differ significantly between the temperature groups (*p* > 0.05) (Table 3).

**Table 3:** Meat quality.

Item	Group				SEM	*p*
	T20	T23	T26	T29		
pH	6.33	6.34	6.36	6.39	0.03	0.949
Luminance L*	34.8	36.2	34.3	35.0	1.3	0.184
Redness a*	17.3	16.8	17.5	16.8	0.3	0.628
Yellowness b*	0.30^b^	0.09^b^	1.29^a^	0.84^ab^	0.25	0.029
Shear force (N)	56.4	58.3	64.8	64.1	0.2	0.123
Cooking loss (g)	17.3^a^	16.9^a^	17.2^a^	12.7^b^	0.6	0.017
Protein (g)	21.0	21.2	22.3	22.2	0.2	0.077
crude fat (g)	1.26	1.53	1.18	1.69	0.07	0.638
Ash (g)	1.91	1.51	1.74	1.79	0.15	0.234

^a,b,c^Means with different superscripts within the same row differ significantly (*p* < 0.05). No letter indicates that the differences are not significant (*p* > 0.05). Results are means with *n* = 9 per treatment.

### 3.4 Serum antioxidant function

The T-AOC was significantly higher in the T29 compared to the other groups (*p* < 0.05), the SOD in the T29 was significantly lower than T23 and T26 (*p* < 0.05). The GSH-PX and MDA were not significantly different between the temperature groups (*p* > 0.05) (Table 4).

**Table 4:** Serum antioxidant function.

Item	Group				SEM	*p*
	T20	T23	T26	T29		
GSH-PX (U/mL)	883.4	850.7	824.4	765.5	25.2	0.415
MDA (nmol/mL)	4.39	4.94	5.53	5.75	0.33	0.433
T-AOC (U/mL)	0.36^b^	0.38^b^	0.38^b^	0.49^a^	0.02	0.021
SOD (U/mL)	26.0^ab^	23.6^b^	22.5^b^	32.4^a^	1.4	0.04

^a,b,c^Means with different superscripts within the same row differ significantly (*p* < 0.05). No letter indicates that the differences are not significant (*p* > 0.05). Results are means with *n* = 9 per treatment.

## 4 Discussion

In our research, the mortality rate was remarkably low. Throughout the entire rearing period, each group had only 2–3 ducks that died (Supplementary Table S1). It’s a common phenomenon in the production of meat ducks and does not impact the experimental results.

Commercial poultry breeds are fast-growing animals that rely on evaporative heat loss to regulate their body temperature, and temperature changes affect feed intake, growth and thermoregulation (26, 37, 38). Increased temperature leads to a decline in the production performance of Pekin ducks. From the results of this experiment, there is a negative correlation between temperature and the market weight of Pekin ducks (Supplementary Table S2). This finding was supported by previous research on chickens (21, 38, 39), ducks (28, 40) and geese (26). And the results of ADFI also followed the pattern of BW changes. However, there was no significant difference in F/G, which was one of the reasons consistent with the trends of ADFI and ADG. This suggested that within the range of 20–29°C, there was no variation in feed efficiency for Pekin ducks. This implied that the body weight of Pekin ducks is almost entirely determined by feed intake. Changes in ambient temperature alter the thermoregulation of Pekin ducks, requiring them to regulate the generation of metabolic heat by adjusting feed intake. The range of 20–29°C did not impact the digestive capacity of Pekin ducks, but this result may be challenged when the temperature exceeds 29°C (26, 28).

Additionally, we observed an increased trend in BW reduction when the temperature exceeded 23°C. This indicated that the suitable temperature range for the growth of Pekin ducks was not a fixed value but rather a temperature interval. When the temperature exceeded the upper critical limit, the BW decreased rapidly. Many studies had also confirmed the existence of an upper critical temperature in duck farming and estimated it using models, including other poultry (26, 28, 40). Homeothermic animals can regulate body temperature within a certain range, and damage occurs once it exceeds the body’s regulatory capacity. This is the reason for the existence of critical temperature. Sun et al.’s study suggested that the upper critical temperature for Pekin ducks was 27.4°C, while Xie et al.’s result was 31.3°C (28, 40). The possible reason for this difference was the different ages of ducks in the two studies, indicating that the ability of ducks to regulate body temperature changed with age, and there were different critical temperatures at different growth stages. In this study, the age of the ducks was consistent with Sun et al., and we found that the upper limit critical temperature was less than 27.4°C after we fed the results into his model. The potential cause of this outcome may be attributed to variations in humidity. In the study conducted by Sun et al., the environmental humidity was set at 60%, while in our research, the humidity was maintained at 74%. This difference could be a significant factor contributing to the leftward shift of the upper critical temperature coordinates in the model.

Genetic factors, age, sex, nutritional level, growth environment and other factors affect body size traits and slaughter traits (23). Body size traits are phenotypic characteristics that respond to the growth and development of the animal, and the measurement of body size traits may be used to select favorable traits and examine the high-quality genetic factors of a breed. The body conformation and slaughter traits of animals are highly correlated with production performance. In this study, the body oblique length, keel length, and chest depth of ducks decreased with increasing temperature, and these indicators showed a consistent trend with changes in BW and ADFI. The potential cause of this outcome may be attributed to temperature influencing the feed intake of ducks, thereby influencing the body development of ducks. Organ indices are important indicators reflecting organ growth and development, directly affecting the nutritional absorption efficiency, metabolism, and growth rate of the organism. Many studies have shown that the environment and feeding levels have a significant impact on organ development (41). The spleen is an important immune organ in ducks, and the liver is an organ for lipid metabolism in the body. Their indices represent the strength of the corresponding functions. In this study, although there were significant differences in liver weight and spleen weight, there were no significant differences in the corresponding ratios. In view of this, the temperature range of this experiment may not have a direct impact on the metabolism and immune function of duck bodies. In this study, there were significant changes in pectoral muscle weight and thigh muscle weight, but there were no significant differences in the corresponding relative weigh. The probable cause for this phenomenon is that the temperature range in this experiment did not impact the meat production efficiency of ducks. However, Zhang et al. (20) found that chronic high temperature reduced the proportion of breast muscles in chickens and increased the proportion of thigh muscles, which was inconsistent with the results of this study. Nevertheless, there was no mention of the relative humidity during the experiment in his paper, making it difficult for us to ascertained if this variation is attributable to relative humidity.

Meat quality is influenced not only by genetic factors but also by environmental factors such as temperature, humidity, light, and harmful gas content. The indicators for evaluating meat quality include pH, meat color (a*, b*, and L* values), shear force, dripping loss, cooking loss, fat, ash and protein (17, 23, 42, 43). Currently, research results on the cooking loss of Pekin ducks under different temperatures are inconsistent. In this study, cooking loss was significantly reduced with the increase of temperature, and the study by Zhang et al. supported the results of this research (20). In this environment, the amount of water and other nutrients lost in the muscle decreased to maintain higher water-holding capacity. However, there was no significant difference in cooking losses in Sun’s study (27). The reasons for this difference may be the different temperature ranges between this study and that of Sun et al., and there may be interactive effects between different relative humidity and temperature, but this has not been clearly reported. There was evidence that cooking loss was negatively correlated with shear force, but the results of this study have not confirmed it (44).

Changes in muscle pH reflect the rate and intensity of glycolysis in muscle, and elevated muscle acidity values affect muscle protein denaturation, meat color differences and tenderness (45). Meat color is used as a visual sensory measure of meat quality and freshness. The results of current studies on meat color and pH are mixed. One study reported reduced L*, b* and pH of pectoral muscle after heat stress treatment for 24 h (46). Compared with normal temperature, high temperature feeding significantly increased the L* and b* of breast meat, and significantly reduced a*, white meat and water seepage (20). In this study, there was no significant difference in pH value, which is consistent with the results of Sun et al. However, research suggested that increased physiological stress and physical activity of animals during transportation or prior to slaughter leadeed to depletion of muscle glycogen and an increase in pH, which result in dark, firm and dry meat (DFD) (47). There was a significant difference in b* values in this study, but the numerical values deviated significantly from the results of other studies, and the data dispersion was high, possibly due to measurement errors. Therefore, the results may not accurately reflect the real situation. The nutritional value of muscles is primarily evaluated by the content of water, protein, fat and ash. Protein determines the nutritional value of meat, and fat is the main carrier of meat flavor. Adequate fat in muscles is of great significance to the palatability and flavor of meat. In this study, there was no significant difference in protein, fat, and ash content. These results indicated that the temperature range in this study did not affect the nutritional value of muscles.

The antioxidant system is the body’s defense system against free radical damage, which is closely related to cell differentiation and tissue development *in vivo*. Maintaining redox balance is essential for effective immunity and health. Therefore, the determination of antioxidant indices may reflect the pattern of free radical metabolic changes *in vivo* (15, 23, 48–50). There are differences in the sensitivity of various antioxidant indicators in the serum of experimental ducks to temperature treatments. In this study, the T-AOC content in the serum of T29 significantly increased. And it was found that SOD activity significantly increased when the temperature exceeded 26°C. This result may be attributed to the increased demand for the body’s antioxidant defense against oxidative damage due to the rise in temperature, a notion supported by the study of Niu et al. (51). Reports indicated that acute heat stress treatment significantly increased SOD levels, and with increasing treatment intensity (52), SOD activity showed a gradient increase (53). This suggests that, under the premise of 74% relative humidity, the temperature range of 20–26°C may be conducive to reducing oxidative damage in Pekin ducks.

In this experiment, the method used to adjust environmental parameters in the artificial environmental control room involves blowing in freshly heated or cooled air. Sensors are positioned around the room, and every 5 seconds, they report to the control center. The new air is introduced only when the average temperature of all sensors reaches the target temperature, without any preprocessing. This may result in temperature deviations from the set value at different locations within the room and a lag in control. Consequently, influenced by various factors, the temperature fluctuates around the set value, creating a dynamic equilibrium. The impact of this on the experimental results is minimal and unavoidable. In addition, we have some challenges in determining the quality of meat. For example, the time it takes for each sample to be removed from the carcass until measured cannot be guaranteed to be exactly the same, which may have some impact on meat color. The insertion position and Angle of the pH meter may also produce errors in the test results. It is worth considering how these potential influencing factors can be further reduced in future studies. Despite this, the results obtained in this experiment remain highly meaningful.

## 5 Conclusion

In conditions with a relative humidity of 74%, temperature significantly affects the growth performance, slaughtering traits, muscle quality and antioxidant function of Pekin ducks. When the ambient temperature exceeded 26°C, the body weight and feed intake of Pekin ducks significantly decreased, and the development of body size and muscle weight followed this pattern. At ambient temperatures of 20°C and 23°C, the growth development and serum redox status of Pekin ducks were more ideal and stable. In specific relative humidity conditions, the temperature conducive to the growth of Pekin ducks may be a range rather than a fixed value. This study can offer data support for livestock production until more in-depth research is demonstrated. In summary, at 74% relative humidity, the suitable growing temperature for Pekin duck is 20–23°C.

## Data availability statement

The original contributions presented in the study are included in the article/Supplementary material, further inquiries can be directed to the corresponding author.

## Ethics statement

The animal study was approved by Animal Ethics Committee of Jiangsu Academy of Agricultural Sciences. The study was conducted in accordance with the local legislation and institutional requirements.

## Author contributions

CX: Conceptualization, Data curation, Methodology, Validation, Writing – original draft, Writing – review & editing. DS: Investigation, Methodology, Writing – original draft, Writing – review & editing. YL: Investigation, Methodology, Writing – original draft, Writing – review & editing. ZP: Data curation, Formal analysis, Writing – original draft, Writing – review & editing. ZD: Data curation, Formal analysis, Writing – original draft, Writing – review & editing. FC: Data curation, Formal analysis, Writing – original draft, Writing – review & editing. RG: Validation, Writing – original draft, Writing – review & editing. RC: Validation, Writing – original draft, Writing – review & editing. ZS: Supervision, Writing – original draft, Writing – review & editing. SY: Conceptualization, Funding acquisition, Project administration, Writing – original draft, Writing – review & editing.
